# Peer distribution of HIV self-test kits to men who have sex with men to identify undiagnosed HIV infection in Uganda: A pilot study

**DOI:** 10.1371/journal.pone.0227741

**Published:** 2020-01-23

**Authors:** Stephen Okoboi, Oucul Lazarus, Barbara Castelnuovo, Mastula Nanfuka, Andrew Kambugu, Andrew Mujugira, Rachel King

**Affiliations:** 1 Infectious Diseases Institute, College of Health Sciences, Makerere University, Kampala, Uganda; 2 Clarke International University, Kampala, Uganda; 3 The AIDS Support Organization (TASO), Kampala, Uganda; 4 School of Public Health, Makerere University, Kampala, Uganda; 5 Department of Global Health, University of California San Francisco, San Francisco, California, United States of America; David Geffen School of Medicine at UCLA, UNITED STATES

## Abstract

**Introduction:**

One-in-three men who have sex with men (MSM) in Uganda have never tested for HIV. Peer-driven HIV testing strategies could increase testing coverage among non-testers. We evaluated the yield of peer distributed HIV self-test kits compared with standard-of-care testing approaches in identifying undiagnosed HIV infection.

**Methods:**

From June to August 2018, we conducted a pilot study of secondary distribution of HIV self-testing (HIVST) through MSM peer networks at The AIDS Support Organization (TASO) centres in Entebbe and Masaka. Peers were trained in HIVST use and basic HIV counselling. Each peer distributed 10 HIVST kits in one wave to MSM who had not tested in the previous six months. Participants who tested positive were linked by peers to HIV care. The primary outcome was the proportion of undiagnosed HIV infections. Data were analysed descriptively.

**Results:**

A total of 297 participants were included in the analysis, of whom 150 received HIVST (intervention). The median age of HIVST recipients was 25 years (interquartile range [IQR], 22–28) compared to 28 years IQR (25–35) for 147 MSM tested using standard-of-care (SOC) strategies. One hundred forty-three MSM (95%) completed HIVST, of which 32% had never tested for HIV. A total of 12 participants were newly diagnosed with HIV infection: 8 in the peer HIVST group and 4 in the SOC group [5.6% vs 2.7%, respectively; P = 0.02]. All participants newly diagnosed with HIV infection received confirmatory HIV testing and were initiated on antiretroviral therapy.

**Conclusion:**

Peer distribution of HIVST through MSM networks is feasible and effective and could diagnose more new HIV infections than SOC approaches. Public health programs should consider scaling up peer-delivered HIVST for MSM.

## Introduction

The Joint United Nations Program on HIV/AIDS (UNAIDS) estimates that 25% of people infected with HIV globally did not know their HIV status in 2018 [1]. Nearly half (47%) of new HIV infections globally occur among key populations and their sexual partners, including men who have sex with men (MSM) [2]. Globally, the risk of HIV acquisition was 28 times higher among MSM than heterosexual men in 2017 [1, 3]. In Uganda, HIV prevalence among MSM is 13.2% compared to 5.9% in the general population and key populations including MSM account for more than a third of new HIV infections [3–6].

HIV testing is the entry point to HIV care; increasing testing coverage is a key step to reaching the first UNAIDS 90-90-90 target, i.e., 90% of people living with HIV knowing their HIV status. However, testing uptake is still low among MSM despite efforts to scale up prevention services for key populations (KP) in Uganda [5–8]. A recent bio-behavioral survey of Ugandan MSM found that 35% had never tested for HIV [6]. HIV self-testing (HIVST), in which an individual performs and interprets his/her own HIV test [8–13], is an acceptable and innovative approach that could increase knowledge of HIV sero-status among MSM, and is recommended by the World Health Organization and Uganda Ministry of Health [3,14–22]. In addition, HIVST is convenient since individuals can test at any time, in private, without having to present to a clinic during fixed working hours. In Uganda, healthcare stigma, discrimination, homophobic violence and criminalization are significant barriers to uptake of facility-based HIV testing among MSM [4, 6, 8, 23]. Scaling up access to HIVST could increase testing coverage among MSM with low access to facility-based services [3, 6]. Studies in other settings have demonstrated that 82–100% of MSM who test positive after HIVST seek additional testing and more than 80% were linked to treatment [11, 24]. However, few studies have evaluated uptake of HIVST among MSM in sub-Saharan Africa [22, 24]

Novel, effective strategies are urgently needed to increase coverage of HIV testing services among MSM, and promote linkage to prevention, care and treatment services. Peer distribution of HIVST kits in MSM networks may increase access to HIV testing in Uganda where 33% of MSM had never tested for HIV in 2012 [8, 9, 12, 25]. In a recent pilot intervention at the AIDS Support Organization (TASO) secondary distribution of referral coupons by MSM peers increased uptake of facility-based HIV testing [26]. Of 200 coupons distributed, 150 MSM (75%) presented at the facility for HIV testing, and >80% were frequent testers [27], suggesting that use of peer strategies could increase MSM uptake of HIV testing and other prevention services.

This study aimed to evaluate the preliminary effectiveness of peer distribution of HIVST oral fluid self-test kits to MSM networks in identifying undiagnosed HIV infection compared with standard-of-care (SOC) approaches.

## Methods

### Study design

Between June and August 2018, we conducted a pilot study of peer-based distribution of HIVST among two MSM cohorts at TASO centres in Masaka and Entebbe, Uganda. We identified MSM peers with the support of staff members responsible for key population programming. Peers signed a confidentiality agreement and verbally consented to distribute the HIVST kits. We compared the preliminary effectiveness of an MSM peer HIVST kit distribution strategy (intervention) with the standard TASO MSM HIV testing approach that consisted of moonlight testing to identify undiagnosed HIV infections. A “moonlight testing approach” is HIV testing during evening hours in MSM hotspots using conventional HIV testing methods. As this was a cross-sectional study, we did not randomize participants to testing strategy.

#### Study sites and MSM HIV testing approaches

TASO is the largest and oldest indigenous non-governmental HIV care provider in sub-Saharan Africa. It was founded in 1987 by a group of people living with, or deeply affected, by HIV/AIDS in order to provide psychosocial support and basic medical care to PLWH. TASO Entebbe and Masaka are two of the 11 TASO HIV care centres of excellence located in Central region of Uganda. By June 2018, TASO Entebbe and Masaka had active client populations of >6,000 and >8,000, respectively [26]. Since 2016, TASO has implemented the Prevention, Care and Treatment (PRECAT) model in which hotspots and peers were identified through civil society partnerships. A snowballing approach was used to distribute coupons inviting MSM to hotspots for conventional HIV testing. All participants who tested between January and March 2018 were included in the comparison group.

#### Study participants and HIVST kit distribution

Fifteen MSM peers from TASO Masaka (7 peers) and TASO Entebbe (8 peers) were trained in HIVST testing procedures and results interpretation by a certified HIVST trainer and a Ministry of Health HIVST focal person. We used the OraQuick HIV test, an approved single-use, qualitative immunoassay to detect antibodies to Human Immunodeficiency Virus Type 1 and 2 (HIV-1/2) in human oral fluid specimens (OraSure Technologies, Bethlehem, PA, USA). Each kit was pre-packed with written and pictorial usage instructions in English and 2 local languages (Luganda and Kiswahili). The instructions clearly showed study participants how to perform an oral fluid-based self-test, the waiting time before reading results, and how to interpret the results.

Peers received training in basic HIV counselling from two certified senior HIV counsellors from TASO and the Infectious Diseases Institute (IDI). Course content included: 1) how to introduce HIVST to MSM, 2) how to troubleshoot conflicting situations that may arise during the HIVST kit distribution, 3) counselling, 4) confirmatory testing, 5) how to address any misunderstanding resulting from test results, and 6) linkage to HIV services. Peers were given 10 HIVST kits each for distribution to MSM in their social and sexual networks in a single wave by targeting those who had not tested in the previous six months and linking participants who tested positive for confirmatory testing and to HIV care services.

To ensure comprehension, peers demonstrated how to perform a self-test using the pictorial instructions included in each kit. The participants were neither required to self-test in the presence of the peer nor to disclose their tests results if they did not want to. Kit recipients were followed-up through phone calls and meeting at hotspots after 1–2 weeks to assess how many had used the kits, how many declined to use HIVST, and establish reasons for HIVST refusal. Peers returned any used or unused HIVST kits to the KP focal person at the TASO facility. In return, they received a transport reimbursement of $3 for each distributed and returned kit. In addition, they received T-shirts, backpacks and umbrellas as tokens of appreciation.

Participants who tested HIV positive were linked to TASO for confirmatory HIV testing using the Uganda HIV testing algorithm. Positive results from confirmatory testing were used to link the participant to antiretroviral treatment (ART) [21, 28]. The peers physically escorted the identified HIV positive participants for a confirmatory test and treatment at TASO since all the eight disclosed their HIV positive status to the peer after HIVST. Self-test devices indicating positive results were independently read by a health worker at the TASO facility and/or peer for quality assurance purposes.

#### Data collection and analysis

Peers provided weekly updates to the TASO KP focal person including accountability of the serialized HIVST kits. Peers were trained to in data collection tools and collected basic socio-demographic data, post- HIV test assessment data and HIV testing history of the kit recipients using a simplified Ministry of Health HIV testing data collection tool. Data was entered in an excel and exported to STATA for analysis.

The primary outcome was yield—the proportion of undiagnosed HIV infections. We described participant characteristics using descriptive statistics. Continuous variables were compared using an unpaired t-test or Wilcoxon rank sum test if not normally distributed. Pearson chi-square tests were used to compare categorical variables. Statistical analyses were performed using STATA, version 15.0 (StataCorp LLC, College Station, TX, USA).

#### Ethical approval

This study was approved by Infectious Diseases Institute Scientific Review Committee, TASO Research Ethics Committee, The University of California, San Francisco Ethics committee, and the Uganda National Council for Science and Technology (UNCST). The English or Luganda verbal informed consent tool approved by the TASO Research Ethics Committee and UNCST was explained to the participant. Study purpose and procedures were explained to all participants. Those who agreed to take part in the study provided verbal consent that was not recorded, consistent with guidelines from regulatory bodies concerned about the criminalization of MSM in Uganda [29].

## Results

### Population characteristics

A total of 297 participants were included in the analysis of which 150 (51%) were accrued using the peer HIVST distribution strategy. Of these, seven participants did not use HIVST. The median age of the remaining 143 participants was 25 years (interquartile range [IQR], [22–28] with a median number of eight sexual partners in the prior year (IQR 4–19) **(Table 1).** Most (58%) had received primary education, while 18% had no formal education. Before receiving their HIVST kit, the majority (82%) of participants received individual pre-HIV counselling and demonstration of HIVST use before accepting the kit and their testing history elicited. One third (32%) had never tested. Of those with history of HIV testing, 91% had tested for HIV only once in their lifetime. Most of the intervention participants (64%) did not know their sexual partner(s) HIV status.

**Table 1:** Demographic characteristics.

Variable	Peer HIVST distribution (N = 143) n (%)	Standard of care HIV testing (N = 147) n (%)
Age in years, median (IQR)	25 (22–28)	28 (25–35)
Education level		
No formal education	53 (37)	1 (5) ^+^
Primary	26 (18)	10 (57)
O-level	17 (12)	6 (33)
A-level	31 (22)	1 (5)
Tertiary and University	17 (12)	
Marital status		
Married	15 (10)	43 (31)
Separated	3 (2)	9 (6)
Widower	3 (2)	1(1)
Single	123 (85)	86 (62)
Counselling mode		
Individual	118 (82)	144 (99)
Couple	15 (10)	2 (1)
Group	11 (8)	00
Received pretest counselling		
Yes	135 (93.8)	139 (96)
No	8 (6.2)	8 (4)
Tested before		
Yes	98 (68)	106 (79)
No	45 (40)	41 (21)
Number of times ever tested		
One	89 (91)	3 (27) ^++^
Two	8 (8)	8 (73)
Three	1 (1)	0
Number of sexual partners, median	8 (4–19)	10 (5–24)
Spouse/partner tested		
Yes	43 (30)	43 (44)
No	9 (6)	26 (27)
Don’t Know	92 (64)	29 (30)
If Yes, n (%)		
Positive	2 (5)	0^+++^
Negative	32 (74)	38 (95)
Don’t Know	9 (21)	2 (5)

Some subtotals in the standard of care arm do not add up to 147.

^+^129 missing values

^++^136 missing values

^+++^107 missing values

#### Peer-distributed HIVST uptake

Peers distributed a total of 150 HIVST kits. The majority (95%) of the participants completed HIVST; 71 in TASO Masaka and 72 in Entebbe **(S1 Fig).** Nearly all (94%) received post-test HIV counselling from peers who distributed the kits or from peers at the TASO health facility. Most (61%) participants tested alone while 39% tested with the support and presence of the peer. Of the participants tested, the majority (85%) trusted the HIVST results. Overall, eight participants tested HIV positive 2/71 (2.8%) from Masaka and 6/72 (8.3%) from Entebbe), and all eight disclosed their HIV test results to the peers. All were linked to care by peers, re-tested to confirm their HIV status and initiated on ART. A total of 147 MSM in the TASO testing program were tested using SOC approaches yielding 4 (2.7%) undiagnosed infections between January and March 2018. In contrast, the peer HIVST kit distribution strategy reached 143 MSM and identified 8 participants with undiagnosed HIV infection during the 3 months of follow-up (5.6% vs 2.7%; P = 0.02, by the Fisher exact test).

## Discussion

Our study found that one-in-three MSM in Uganda had never tested for HIV. Peer HIVST distribution identified twice as many undiagnosed HIV infections as SOC testing approaches. In this pilot study, that included 297 MSM, peer distributed HIVST reached significantly more never testers or MSM who had not tested in the previous six months. All participants who tested positive were linked for confirmatory testing and HIV care through appointment. Participants who did not turn up for appointments were followed up by peers, accompanied to the HIV care centre for a confirmatory test, and started on ART.

To our knowledge, this is one of the first studies in sub-Saharan Africa to evaluate secondary distribution of HIVST by MSM peers to their social and sexual networks. Our findings suggest that distributing HIVST kits through peer networks is feasible, acceptable and could be an effective strategy to increase uptake of HIV testing and linkage to care in a marginalized and stigmatized population with low testing coverage [8–15]. Our results are in agreement with prior studies done among heterosexual fishing communities in Uganda, and MSM in the United Kingdom and United States, in which peer distribution of HIVST had high uptake and acceptability and successfully identified undiagnosed HIV infections [13,18, 30, 31]. Identifying new HIV infections is an important first step to optimizing the treatment and care cascade for people infected with HIV, particularly for African MSM who are disproportionately affected by the HIV epidemic. The ability of the peer HIVST distribution strategy to reach MSM never-testers or infrequent testers suggests that it could increase testing coverage by accessing MSM who have never tested or tested once in life time and help attain the first UNAIDS 90 target—90% of HIV-infected MSM knowing their status.

Our findings support growing evidence that peer-based strategies are effective in increasing HIV testing among high-risk populations including MSM [13, 18]. A recent study compared respondent driven sampling to conventional outreach-based HIV testing strategies and found that peer-based chain recruitment was more efficient than conventional testing and counseling at identifying HIV infections among MSM [30]. However, prior studies that assessed peer-based testing strategies typically required testers to present at a clinic or research site for an HIV test, and thus only partially address the social and structural barriers that minority MSM encounter when seeking an HIV test. Community-based HIV self-testing delivered by members of a target community or social group could be more effective in reaching those who may not seek testing in high-stigma contexts [20, 32]. This approach builds on prior experiences of peer-driven distribution of HIV prevention tools and referral coupons for HIV testing in key populations, e.g., coupon distribution among MSM in Uganda [8–12].

The strengths of our study include leveraging peer social and sexual networks to increase HIV testing coverage through secondary distribution of HIVST. While we were unable to determine if peer recruiters and those that tested were in the same sexual networks, closed or interconnected sexual networks might help explain high HIV prevalence among MSM in this setting. A limitation of our study was that we report non-randomized comparisons which should be interpreted with caution. Few MSM tested positive so we may be overstating the yield of our peer-delivered intervention. Nevertheless, other studies support the effectiveness of peer-delivered combination prevention. Our results may not be generalizable to all MSM, because our sample was largely comprised of younger MSM. However, these findings contribute to the evidence base for African MSM, an understudied population. Information on education and HIV testing history was not collected during routine TASO program activities and there was considerable missing data. Nevertheless, missing data did not affect ascertainment of the primary outcome. Finally, we did not debrief peers to ascertain their experiences with HIVST distribution or assess whether they would distribute HIVST kits in the future, which could inform future program implementation. The time period for HIVST kits distribution was different from the standard of care moonlight and hotspot approaches, and it is possible that temporal trends confounded study outcomes.

## Conclusion

We found that distributing HIVST kits through MSM peer-networks was feasible, acceptable and effective and could increase uptake of HIV testing and reduce undiagnosed infections among MSM in Uganda. Future implementation research should determine the cost-effectiveness of this strategy in finding and linking persons with undiagnosed infections to HIV care.

## Supporting information

S1 Fig Study profile. (DOCX)

## References

[1] UN Joint Programme on HIV/AIDS (UNAIDS). Global AIDS Update: Miles to go—closing gaps, breaking barriers, righting injustices. Report [Internet]. 2018;268. Available from: http://www.unaids.org/sites/default/files/media_asset/miles-to-go_en.pdf

[2] UNAIDS. The GAP Report. 2014;1–422. Available from: http://www.unaids.org/en/media/unaids/contentassets/documents/unaidspublication/2014/UNAIDS_Gap_report_en.pdf

[3] Wong V, Jenkins E, Ford N, Ingold H. To thine own test be true: HIV self‐testing and the global reach for the undiagnosed. J Int AIDS Soc [Internet]. 2019;22(S1):e25256 Available from: https://onlinelibrary.wiley.com/doi/abs/10.1002/jia2.25256 30912306 10.1002/jia2.25256 PMC6433601

[4] UNAIDS. Uganda | 2017. Country Factsheets. 2017.

[5] Uganda AIDS Commission. The National HIV and AIDS indicator handbook. Republic of Uganda; Uganda AIDS Commission. 2015;(April 2015). Available from: http://www.aidsuganda.org

[6] Hladik W, Sande E, Berry M, Ganafa S, Kiyingi H, Kusiima J, et al Men Who Have Sex with Men in Kampala, Uganda: Results from a Bio-Behavioral Respondent Driven Sampling Survey. AIDS Behav. 2017;21(5):1478–90. 10.1007/s10461-016-1535-2 27600752

[7] Matovu JKB, Musinguzi G, Kiguli J, Nuwaha F, Mujisha G, Musinguzi J, et al Health providers’ experiences, perceptions and readiness to provide HIV services to men who have sex with men and female sex workers in Uganda—A qualitative study Public Health and Health Services. BMC Infect Dis. 2019;19(1). 10.1186/s12879-019-3713-0 PMC6400025 30832612

[8] Okoboi S, Twimukye A, Lazarus O, Castelnuovo B, Agaba C, Immaculate M, et al Acceptability, perceived reliability and challenges associated with distributing self‐test kits to young MSM in Uganda: a qualitative study. J Int AIDS Soc [Internet]. 2019;22(3):e25269 Available from: https://onlinelibrary.wiley.com/doi/abs/10.1002/jia2.25269 30932364 10.1002/jia2.25269 PMC6441924

[9] Lightfoot MA, Campbell CK, Moss N, Treves-Kagan S, Agnew E, Kang Dufour MS, et al Using a social network strategy to distribute HIV self-test kits to African American and Latino MSM. In: Journal of Acquired Immune Deficiency Syndromes. J Acquir Immune Defic Syndr. 2018 9 1;79(1):38–45 10.1097/QAI.0000000000001726 29771792

[10] Lippman SA, Lane T, Rabede O, Gilmore H, Chen YH, Mlotshwa N, et al High Acceptability and Increased HIV-Testing Frequency After Introduction of HIV Self-Testing and Network Distribution Among South African MSM. J Acquir Immune Defic Syndr. 2018;77(3):279–87. 10.1097/QAI.0000000000001601 29210826 PMC5807184

[11] Zhong F, Tang W, Cheng W, Lin P, Wu Q, Cai Y, et al Acceptability and feasibility of a social entrepreneurship testing model to promote HIV self-testing and linkage to care among men who have sex with men. HIV Med. 2017;18(5):376–82. 10.1111/hiv.12437 27601301 PMC5340630

[12] Sharma A, Chavez PR, MacGowan RJ, McNaghten AD, Mustanski B, et al Willingness to distribute free rapid home HIV test kits and to test with social or sexual network associates among men who have sex with men in the United States. AIDS Care—Psychol Socio-Medical Asp AIDS/HIV. 2017;29(12):1499–503. 10.1080/09540121.2017.1313386 PMC12186660 28393612

[13] Choko AT, Nanfuka M, Birungi J, Taasi G, Kisembo P, Helleringer S. A pilot trial of the peer-based distribution of HIV self-test kits among fishermen in Bulisa, Uganda. PLoS One. 2018;13(11). 10.1371/journal.pone.0208191 PMC6264512 30496260

[14] Volk JE, Lippman SA, Grinsztejn B, Lama JR, Fernandes NM, Gonzales P, et al Acceptability and feasibility of HIV self-testing among men who have sex with men in Peru and Brazil. Int J STD AIDS. 2016;27(7):531–6. 10.1177/0956462415586676 25971262 PMC4643427

[15] Figueroa C, Johnson C, Verster A, Baggaley R. Attitudes and Acceptability on HIV Self-testing Among Key Populations: A Literature Review. AIDS Behav. 2015;19(11):1949–65. 10.1007/s10461-015-1097-8 26054390 PMC4598350

[16] Wood BR, Ballenger C, Stekler JD. Arguments for and against HIV self-testing. HIV/AIDS—Res Palliat Care. 2014;6:117–26. 10.2147/HIV.S49083 PMC4126574 25114592

[17] Young SD, Daniels J, Chiu CJ, Bolan RK, Flynn RP, Kwok J, et al Acceptability of using electronic vending machines to deliver oral rapid HIV self-testing kits: A qualitative study. Vol. 9, *PLoS One*. 2014 7 30;9(7): e103790 10.1371/journal.pone.0103790 25076208 PMC4116256

[18] Wong V, Johnson C, Cowan E, Rosenthal M, Peeling R, Miralles M, et al HIV self-testing in resource-limited settings: regulatory and policy considerations. AIDS Behav. 2014;18 Suppl 4(PG-S415-21):S415–21. 24957979 10.1007/s10461-014-0825-9

[19] Kalibala S Tun W, Cherutich P, Nganga A, Oweya E, Oluoch P. Factors associated with acceptability of HIV self-testing among health care workers in Kenya. AIDS Behav. 2014;18 Suppl 4: S405–414. 24974123 10.1007/s10461-014-0830-z PMC4933836

[20] Krause J, Subklew-Sehume F, Kenyon C, Colebunders R. Acceptability of HIV self-testing: a systematic literature review. BMC Public Health. 2013;13:735 10.1186/1471-2458-13-735 23924387 PMC3750621

[21] Ng OT, Chow AL, Lee VJ, Chen MI, Win MK, Tan HH, Chua A, Leo YS. Accuracy and User-Acceptability of HIV Self-Testing Using an Oral Fluid-Based HIV Rapid Test. PLoS One. 2012;7(9). 10.1371/journal.pone.0045168 PMC3444491 23028822

[22] Flowers P, Riddell J, Park C, Ahmed B, Young I, Frankis J, et al Preparedness for use of the rapid result HIV self-test by gay men and other men who have sex with men (MSM): a mixed methods exploratory study among MSM and those involved in HIV prevention and care. HIV Med. 2017;18(4):245–55. 10.1111/hiv.12420 27492141 PMC5347967

[23] Musoba N et al The Uganda HIV and AIDS Country Progress Report." Kampala: Uganda AIDS Commission (2016).

[24] Zhang C, Li X, Brecht ML, Koniak-Griffin D. Can self-testing increase HIV testing among men who have sex with men: A systematic review and meta-analysis. PLoS One. 2017;12(11). 10.1371/journal.pone.0188890 PMC5708824 29190791

[25] Choko AT, Taegtmeyer M, Macpherson P, Cocker D, Khundi M, Thindwa D, et al Initial accuracy of HIV Rapid test kits stored in suboptimal conditions and validity of delayed reading of oral fluid tests. PLoS One. 2016;11(6). 10.1371/journal.pone.0158107 PMC4918937 27336161

[26] TASO Website, viewed on 02 January, 2019. https://www.tasouganda.org/

[27] King R, Barker J, Nakayiwa S, Katuntu D, Lubwama G, Bagenda D, et al Men at risk; a qualitative study on HIV risk, gender identity and violence among men who have sex with men who report high risk behavior in Kampala, Uganda. PLoS One. 2013;8(12). 10.1371/journal.pone.0082937 PMC3866199 24358239

[28] Mavedzenge S, Baggaley R, Lo Y, Corbett L. HIV self-testing among health workers Africa South of the Sahara.World Health Organization 2011.

[29] Uganda National Council for Science & Technology. National guidelines for research involving humans as research participants; 2014.

[30] Thirumurthy H, Masters SH, Mavedzenge SN, Maman S, Omanga E, Agot K. Promoting male partner HIV testing and safer sexual decision making through secondary distribution of self-tests by HIV-negative female sex workers and women receiving antenatal and post-partum care in Kenya: a cohort study. Lancet HIV. 2016;3(6):e266–74. 10.1016/S2352-3018(16)00041-2 27240789 PMC5488644

[31] Samuel H. Masters, Kawango Agot, Beatrice Obonyo, Sue Napierala Mavedzenge, Suzanne Maman, Harsha Thirumurthy. Promoting Partner Testing and Couples Testing through Secondary Distribution of HIV Self-Tests: A Randomized Clinical Trial. PLoS Med. 2016;13:e1002166 10.1371/journal.pmed.1002166 27824882 PMC5100966

[32] TASO 2017 annual report. Viewed 03 March, 2019. https://www.tasouganda.org/

