# Supplementary material for: Peer distribution of HIV self-test kits to men who have sex with men to identify undiagnosed HIV infection in Uganda: A pilot study
Source: PLoS One. 2020 Jan 23;15(1):e0227741. doi: 10.1371/journal.pone.0227741 (PMC6977761; doi:10.1371/journal.pone.0227741)
Supplement: S1 Fig — (DOCX) [file pone.0227741.s001.docx]

**S1 Fig: Study Profile**

Study Cohort

(n=297)

Standard of-care Testing (n=147)

Peer HIVST Distribution (n=150)

Completed HIV Test (n=143)

71 MSM Completed HIVST in TASO Masaka

72 Completed the HIVST in TASO Entebbe

4 (2.7%) Tested HIV Positive

6 (8.3%) Tested HIV Positive

2 (2.8%) Tested HIV Positive
